# The Effects of Open Innovation Based on Mergers and Acquisitions on Innovative Behavior of Enterprises: Evidence From Chinese Listed Enterprises

**DOI:** 10.3389/fpsyg.2021.794531

**Published:** 2022-01-20

**Authors:** Min Wu, Tao Luo, Yihao Tian

**Affiliations:** ^1^Department of Public Service Management and Public Policy, School of Public Administration, Sichuan University, Chengdu, China; ^2^Social Development and Social Risk Control Research Center, Sichuan Philosophy and Social Sciences Key Research Base, Chengdu, China

**Keywords:** innovative behavior, overseas mergers and acquisitions, open innovation, independent innovation, difference-in-difference

## Abstract

Finding the factors driving enterprise innovation behavior from multiple dimensions is of great significance for promoting enterprise innovation. Open innovation based on overseas mergers and acquisitions (M&A) has become one of the main ways for enterprises to obtain knowledge and technology. However, there is still no agreement on whether open innovation based on overseas M&A can promote innovation behavior of enterprises. Based on data from M&A transaction and enterprise patent of China’s Shanghai and Shenzhen A-share listed companies from 2011 to 2018, this study constructs a propensity score matching and difference-in-difference model from the perspective of innovation performance and innovation investment empirically studies the influence of open innovation mode based on overseas M&A on the innovation behavior of enterprises and finds that open innovation based on overseas M&A can significantly promote the innovation performance and innovation investment. Meanwhile dynamic effects test shows this promotion effect is sustainable; it reaches the maximum in the year of overseas M&A and decreases in the next two years. In addition, the impacts are heterogeneous due to enterprise ownership and enterprise technology intensity. The findings extends the scope of understanding innovation behavior of enterprises from overseas M&A and provide solid evidence of significant business implications for the promotion of entrepreneurial innovation.

## Introduction

Innovation is widely recognized as the main strategic driving force that leads to economic growth and development (Scuotto et al., 2020). As the main body of innovation, the improvement of an enterprise’s innovation capability is the key to innovation-driven development (Jahanger, 2021). However, the complex interaction between technological paradigm and knowledge flows is making innovation more difficulty and expensive. In a nutshell, it becomes increasingly difficult for enterprises to achieve independent innovation on internal resources alone (Scuotto et al., 2020). The alternative is to search the external resources to gain the chances of innovation and achieve comparative advantages in fierce global competition. This leads firms to an open innovation system. As Chesbrough (2003) proposed in the early 2000s, open innovation means using knowledge inflow and outflow to promote enterprises to speed up internal innovation and broaden the market for the use of external resources such as partnership, licensing contracts, industry-university-research, that is, multiple subjects’ synergetic governance of enterprises, universities and government to promote talent cultivation and technological innovation, and other technology agreements (Duysters and Hagedoorn, 2001; Drayton and Budinich, 2010; Del Giudice and Maggioni, 2014; Carayannis et al., 2018). As one of the main way of open innovation (Berchicci, 2013), in the past ten years, mergers and acquisitions (M&A) have constantly grown (Bresciani, 2012; Öberg, 2017) and become one of the main ways used by firms to obtain knowledge and technology resources for innovation (Öberg, 2016; Shin et al., 2017) and augment their performance (Dezi et al., 2018).

The relationship between M&A and innovation has received attention from both practice and academia, but the conclusions are inconsistent. Some scholars provide evidence that M&A can promote innovation in firms. For example, M&A enables acquiring companies to learn directly from overseas acquired companies and obtain complementary R&D resources, which is conducive to breaking the dependence on technological innovation, changing the company’s innovative thinking, and promoting innovation (Stiebale, 2013). Furthermore, by reconfiguring the knowledge network, providing economies of scale and scope in research, and boosting the capacity for inventive recombination, M&A can enhance the acquirer’s knowledge base and improve its innovation output (Bresciani and Ferraris, 2016; Chen et al., 2021). Conversely, other scholars suggest that M&A has a negative effect on company’s innovation. Specifically, M&A involves managerial problems, integration issues, and transaction expenses (Zollo and Singh, 2004; Del Giudice and Maggioni, 2014; Carayannis et al., 2017). When companies conduct M&A, the cost of integrating and adjusting resources due to cultural systems and other differences leads to technology spillover and suboptimal performance (Edamura et al., 2014). Another viewpoint is that the influence of M&A on company’s independent innovation is unclear (Zhou et al., 2019). The latest empirical evidence suggests that firms completing overseas M&A witness an increase in systemic innovation but a drop in autonomous innovation (Zhang and Tong, 2021).

**FIGURE 1:**
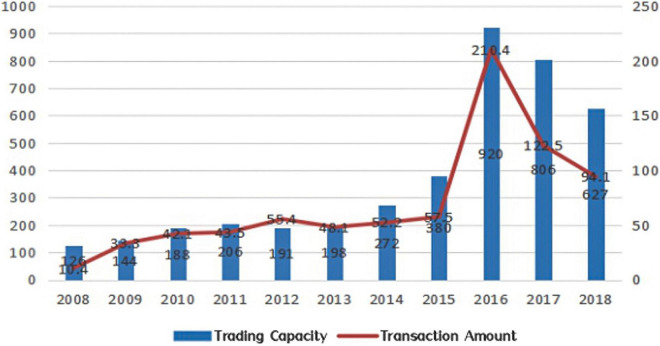
Overseas M&A of Chinese enterprises from 2008 to 2018. Data obtained from enterprise patent database in wind.

Given the above, the relationship between open innovation based on M&A, especially overseas M&A, and the independent innovation behavior of enterprises is still not clear. Whether open innovation based on overseas M&A can promote the independent innovation behavior of enterprises? Furthermore, what is the heterogeneity of overseas M&A in terms of ownership, and technology intensiveness? To answer the above questions, the active overseas M&A of Chinese companies in recent years provide a unique opportunity for this study. Between 2008 and 2018, the number of overseas M&A of Chinese companies increased from 126 to 627, and the amount of M&A increased from $10.4 billion to $94.1 billion, with the number and amount of M&A peaking at 920 in 2016, involving more than $200 billion (see Figure 1).

Specifically, about the methodology, there are many approaches towards studying M&A activities. It is possible to identify three principal streams of study. The empirical quantitative method is often used, the empirical qualitative method (case study/multiple case study) and the desk qualitative method. From the perspective of this study, in order to clearly identify the causal effect of M&A on independent innovation of enterprises, this article adopts the causal inference method in empirical quantitative method. Based on overseas M&A data and patent data of A-share listed companies from 2011 to 2018, this study takes the overseas M&A as a quasi-natural experiment, uses propensity score matching (PSM) to solve the self-selection effect of overseas M&A enterprises and constructs difference-in-difference (DID) from the perspective of innovation performance and innovation investment to study the impacts of open innovation based on overseas M&A on enterprises’ independent innovation empirically. This study finds that open innovation based on overseas M&A can significantly promote the innovation performance and innovation investment. Meanwhile dynamic effects test shows this promotion effect is sustainable; it reaches the maximum in the year of overseas M&A and decreases in the next 2 years. In addition, the impacts are heterogeneous due to enterprise ownership and enterprise technology intensity.

This study may have three contributions to the current literature. First, we use data from developing country to empirically examine the causality between the open innovation based on overseas M&A and independent innovation of listed companies, which provides new evidence for understanding the relationship between overseas M&A and corporate innovation. Second, this study regards the overseas M&A of listed companies as a quasi-natural experiment and uses the DID and PSM methods to solve the sample self-selection bias and reduce the endogenous problem, which clarifies the causal identification clearly. Third, this study considers both the innovation performance and the innovation investment of enterprises innovation behavior and further analyzes the heterogeneous innovation effect of open innovation based on overseas M&A among different enterprise ownership and technology intensity. This study contributes to a comprehensive understanding of the innovation effects of open innovation based on overseas M&A.

## Experimental Details

### Theoretical Analysis and Research Hypothesis

Theoretically, open innovation may have positive or negative effects based on overseas M&A on the independent innovation behavior of enterprises. The positive impact is reflected in the fact that enterprises’ technology and innovation strategies rely more on open innovation, especially overseas M&A (Watanabe et al., 2009). M&A can enable enterprises to quickly acquire high levels of expertise, R&D skills, experienced employees, and specific new technologies to meet the challenges of a dynamic and competitive environment (Bower, 2001). The innovation performance of overseas M&A enterprises is not only increased in quantity, but the quality of innovation is also significantly improved (Hull and Rothenberg, 2008), and the improvement of M&A to long-term innovation ability is more significant than the improvement of short-term innovation ability (Entezarkheir and Moshiri, 2018). The acceleration of innovation and the demand for new solutions are the main factors that drive enterprises to obtain external resources and capabilities through M&A (King et al., 2008). The negative influence is reflected in the fact that enterprises need to spend a significant amount of time and material consumption to integrate after M&A. Meanwhile, the increase in transaction costs also has a negative effect on the development of innovation after M&A (Ahuja and Katila, 2001). M&A absorbs the time and energy of managers and reduces their commitment to long-term investment in R&D, resulting in a decline in innovation performance after M&A (Hitt et al., 1991; Hoskisson et al., 1994). Moreover, when the target enterprises of overseas M&A are in the same industry as the original enterprises, M&A behavior cannot promote the innovation performance of enterprises, which may be due to the difficulty of integration after M&A and the lack of experience (Kreiser et al., 2013). In recent years, the overseas M&A activities of Chinese enterprises have taken place on a large scale, the experience of M&A has gradually accumulated, and the success rate of M&A has greatly improved.

Therefore, according to the actual situation in China, we propose the following hypothesis:

Hypothesis 1: Open innovation based on overseas M&A has a positive impact on the innovation of enterprises, and innovation performance and innovation investment have significantly improved.

The above theoretical analysis emphasizes the role of open innovation based on overseas M&A in promoting enterprise innovation, but different enterprise ownership and whether they are technology-intensive, high-tech enterprises play a heterogeneous role in the innovation effect of open innovation based on overseas M&A (Aghion et al., 2013; Foroughi et al., 2015). Many studies have shown that the innovation efficiency of state-owned enterprises (SOEs) is significantly lower than that of foreign-funded and private enterprises (Laffont and Tirole, 1993; Jefferson et al., 2006). However, as China’s economic growth momentum shifts to innovation-driven, SOEs have started to pay attention to serving the national strategy and enhancing technological innovation capabilities by acquiring knowledge, technology, and resources in overseas M&A (Bierwerth et al., 2015; Hervas-Oliver et al., 2016). This study holds that although the innovation performance and motivation of SOEs are weaker than that of non-SOEs before M&A, after adopting the open innovation model of overseas M&A to obtain advanced technology, their own technological innovation ability will be significantly improved, and their innovation performance will be significantly increased. For non-SOEs, the integration of overseas M&A is more difficulty and risky, and the adaptation time is longer. Compared with SOEs, in order to promote the absorption and transformation of foreign technology, it is necessary to further increase innovation investment and improve the intensity of R&D investment and the proportion of technical personnel (Shefer and Frenkel, 2005). Simultaneously, the expansion of the organizational scale will lead to a reduction in management limitation and an increase in information transmission costs, which will have a negative impact on the innovation performance of non-SOEs. Therefore, after the completion of overseas M&A, the innovation performance of non-SOEs will be less improved than that of SOEs in that year. In order to complete the technological transformation, investment in innovation would still have increased more than that of SOEs.

Therefore, we propose the following hypothesis:

Hypothesis 2: The promotion effect of the open innovation mode based on overseas M&A on the innovation performance of SOEs is more obvious than that of non-SOEs, but the promotion effect of SOEs’ innovation input is weaker than that of non-SOEs.

There are obvious differences in innovation activities between high-tech enterprises and non-high-tech enterprises (Duysters and Hagedoorn, 2001). It is necessary to divide the samples into two subsamples, namely, high-tech enterprises and non-high-tech enterprises, and then explore the impact of open innovation based on overseas M&A on the independent innovation behavior of enterprises. Compared to non-high-tech enterprises, the innovation motive force and innovation ability of high-tech enterprises are obviously stronger and corresponding innovation input and performance are also higher. In this case, the promotion effect of open innovation behavior based on overseas M&A on the innovation performance and investment of high-tech enterprises is weaker than that of non-high-tech enterprises. Meanwhile, high-tech enterprises are based more on technical considerations to carry out overseas M&A. After the M&A of cultural integration, technology integration, and other aspects of higher requirements, it will take longer for integration to be achieved; the risk of M&A failure is greater, and M&A patent performance will be greatly affected (Aminova, 2016). This study holds that open innovation based on overseas M&A plays a greater role in promoting the innovation performance of non-high-tech enterprises than high-tech enterprises.

Therefore, we propose the following hypothesis:

Hypothesis 3: The effect of open innovation based on overseas M&A on innovation performance and investment in non-high-tech enterprises is more obvious than that of high-tech enterprises.

### Research Design

#### Sample and Data

To examine whether open innovation based on overseas M&A promotes the independent innovation behavior of enterprises, this study uses data from State Intellectual Property Office, Wind database and China Stock Market & Accounting Research Database (CSMAR). This study selects the Shanghai and Shenzhen A-share listed companies in China from 2008 to 2018 as the initial sample. The overseas M&A data of listed companies originate from the Wind listed company M&A database (MA), and further confirm the M&A behavior and the information of the M&A party through the listed company announcement. The number of patent applications originates from the State Intellectual Property Office and CSMAR, R&D investment, the proportion of technical personnel, and other data from the Wind database. Simultaneously, the samples of missing data, enterprises with financial industry or ST (Special Treatment. It refers to an enterprise with abnormal financial or other conditions), and less than 10% of acquired shares are removed, and 733 overseas M&A transaction records of listed companies are obtained after preliminary screening. For enterprises with multiple M&A activities in different years, the completion of the first M&A prevail. Since listed companies began to disclose the proportion of technical personnel in 2011, and there were few M&A records before 2011, the final M&A sample is 247 listed companies. Through the above processing, the non-parallel panel data of 24,963 observations of 3,333 enterprises from 2011 to 2018 are finally obtained. Among them, 247 enterprises with overseas M&A are in the treatment group, and 3,086 enterprises without overseas M&A are in the control group.

#### Methods

The question explored in this study is whether open innovation based on overseas M&A promotes the independent innovation behavior of enterprises and is conducive to the high-quality development of enterprises. However, overseas M&A behavior does not occur at random. Only those enterprises with a high level of productivity, who lead industry development, and actively seek innovative technology will choose to invest abroad (Ornaghi, 2009; Mao et al., 2015); overseas M&A may have a “self-selection effect.” It is unreasonable to directly compare the innovation activities of overseas M&A enterprises with those of other enterprises. This study uses the practice of Ornaghi (2009) for reference, regards overseas M&A as a quasi-natural experiment, and adopts the PSM method to solve the self-selection effect of enterprises (Abadie and Cattaneo, 2018). On the basis of matching samples, the DID method is used to measure the impact of open innovation based on overseas M&A on enterprise innovation, which reduces the problem of endogeneity in estimation and provides clearer and more reliable results for causal inference (Doudchenko and Imbens, 2016). The first difference stems from the enterprise level, while the second layer stems from the time series level. Specifically, this study compares the differences between M&A enterprises and matching non-M&A enterprises before and after M&A. The model is defined as follows:


(1) $$Y_{i\,t}=\alpha +\beta \,d\,i\,d_{i\,t}+X_{i\,t}^{{}^{\prime }}\,\varphi +\eta_{j}+\gamma_{t}+\varepsilon_{i\,t}$$

Where *i* is the individual of the enterprise and *t* is the time. *did*_*it*_ is a double difference item, *did*_*it*_=1, which means that enterprise *i* has overseas M&A in year *t*. If there is more than one overseas M&A activity in the sample period, this study defines the time dummy variable only by the date of the first successful overseas M&A announcement.

This study only defines the time dummy variable by the date of the first successful overseas M&A announcement. *Y*_*it*_ is the index of enterprise innovation, including innovation performance and innovation input. This study measures the performance of enterprise innovation from the perspective of patent quantity and quality. Patent is the logarithm of the sum of the invention and utility model patents applied by the enterprise in that year plus 1, which is used to measure the number of patents. Invention is the logarithm of the number of invention patents applied for by the enterprise in that year plus 1, which is used to measure the quality of the patents. For the measurement of innovation investment, this study considers the intensity of R&D investment (Rd) and the proportion of human capital investment—the proportion of technical personnel (Rdp)—to comprehensively and accurately evaluate the impact of overseas M&A on the innovation effect of enterprises. $X_{i\,t}^{{}^{\prime }}$ is a series of individual-year control variables. These variables include enterprise size (Size), asset-liability ratio (Lev), labor productivity (Lap), capital intensity (Capital), financing constraint (Fc), enterprise age (Age), overseas business revenue (Oversea), and enterprise control attribute (State). The variables are shown in Table 1. η_*j*_ refers to the industry fixed effect, which controls all factors at the industry level that do not change with time, such as industry characteristics. γ_*t*_ stands for the time fixed effect, which controls the characteristics of the time level that do not change with the change of enterprises, such as the change in the macroeconomic situation. β is a did regression coefficient through which the influence effect of overseas M&A on enterprise innovation can be judged. In the above estimation formula, this study focuses on the coefficient β, if $\hat{\beta }>0$; that is, compared with the enterprises without overseas M&A, overseas M&A improves the innovation capability of M&A enterprises.

**TABLE 1:** Specific definition of variables.

Variable	Variable definition
**Interpreted variable**	
Patent quantity	Ln (1 + number of invention patents and utility model patents applied by an enterprise in the same year)
Patent quality invention	Ln (1 + number of invention patents applied by an enterprise in the same year)
Research and development investment (Rd)	R&D input intensity; Rd = R&D expenses/operating income
Human capital investment (Rdp)	Proportion of technicians; Rdp = the number of technicians/employees
**Control variable**	
Enterprise size (Size)	The size of the enterprise, expressed by log (total number of employees in that year)
Asset-liability ratio (Lev)	Asset-liability ratio, Lev = total liabilities at the end of the period/total assets at the end of the period
Labor productivity (Lap)	Labor productivity, Lap = log (operating income/total number of employees)
Capital intensity (Capital)	Capital intensity, Capital = fixed asset balance/total number of employees at the end of the period
Financing constraint (Fc)	Enterprise financing constraints, Fc = financial expenses/operating income
Enterprise age (Age)	Number of years of establishment of an enterprise
Overseas business revenue (Oversea)	Overseas business income, greater than 0, is recorded as 1, otherwise it is recorded as 0
Enterprise control attribute (State)	The attribute of enterprise control rights, the state-owned enterprise is recorded as 1, otherwise it is recorded as 0

As can be seen from Table 2, the overall patent performance level of overseas M&A enterprises (both the number and quality of patents) is significantly higher than that of enterprises without overseas M&A. The average number of invention patents and overseas M&A enterprises is about 10.63, while the average number of overseas M&A enterprises is 3.18; the former is about 3.3 times that of the latter. Simultaneously, the average number of invention patent applications of overseas M&A enterprises is about 5.66, while the average number of overseas M&A enterprises is about 1.87; the former is about three times that of the latter, which preliminarily shows that overseas M&A not only helps to improve the innovation performance of enterprises, but also greatly improves the quality of performance.

**TABLE 2:** Descriptive statistics of all variables.

Variable	Mean	Std. Dev	Min	Median	Max	Obs
**All samples**						
Number of patents	1.511	1.794	0.000	0.693	9.743	24,963
Patent quality	1.121	1.499	0.000	0.000	9.168	24,963
Rd	0.038	0.042	0.000	0.032	0.240	24,963
Rdp	0.168	0.179	0.000	0.121	0.827	24,963
Size	7.394	1.308	2.197	7.318	13.021	24,963
Lev	0.421	0.205	0.052	0.412	0.901	24,963
Lap	13.689	0.886	5.825	13.578	19.886	24,963
Capital	12.303	1.252	4.127	12.343	21.335	24,963
Fc	0.015	0.035	–0.063	0.007	0.207	24,963
Age	17.353	6.054	1.000	17.000	64.000	24,963
Oversea	0.572	0.495	0.000	1.000	1.000	24,963
State	0.283	0.450	0.000	0.000	1.000	24,963
**Overseas M&A enterprises**						
Number of patents	2.454	2.035	0.000	2.565	9.743	1,953
Patent quality	1.896	1.821	0.000	1.609	9.168	1,953
Rd	0.038	0.040	0.000	0.033	0.240	1,953
Rdp	0.210	0.189	0.000	0.153	0.827	1,953
**Non-overseas M&A enterprises**						
Number of patents	1.431	1.749	0.000	0.000	9.524	23,010
Patent quality	1.055	1.450	0.000	0.000	8.918	23,010
Rd	0.038	0.043	0.000	0.032	0.240	23,010
Rdp	0.165	0.178	0.000	0.118	0.827	23,010

The overall innovation investment of overseas M&A enterprises is higher than that of enterprises without overseas M&A. In terms of R&D investment intensity, the average of the two is the same; R&D capital investment accounts for less than 4% of business income, indicating that enterprises still do not pay enough attention to R&D capital investment. However, in terms of the proportion of scientific researchers, 21.0% of the enterprises engaged in overseas M&A, while only 16.5% of the enterprises did not carry out overseas M&A; the former was about 3.5 percentage points higher than the latter. It can be preliminarily considered that overseas M&A have effectively promoted the investment of enterprises in scientific researchers, but what should not be ignored is that there are also great individual differences. If accurate results are to be obtained, further empirical tests are needed.

## Results

In this section, we conduct a series of empirical analyses on whether and how open innovation based on overseas M&A contributes to the independent innovation behavior of enterprises. Firstly, we match the overseas and non-overseas M&A enterprises using the PSM method in section “Results of PSM.” Then, we conduct an empirical analysis using the DID method in section “Results of DID.” Finally, we perform a heterogeneity analysis in section “Results of Heterogeneity Analysis.”

### Results of PSM

This study uses the PSM method to match overseas and non-overseas M&A enterprises to ensure the reliability of the matching results. Before using PSM to control the endogeneity of overseas M&A, it is necessary to determine which factors are more likely to lead to overseas M&A (Ornaghi, 2009). Based on the standard proposed by Smith and Todd (2005), this study selects the following variables: enterprise size (Size), asset-liability ratio (Lev), labor productivity (Lap), capital intensity (Capital), enterprise financing constraint (Fc), enterprise age (Age), Overseas business income (Oversea > 0 is marked as 1, otherwise 0), and enterprise nature (State). The Logit model is used to predict the probability of overseas M&A, and the results are shown in Table 3.

**TABLE 3:** Regression results of Logit model.

	Estimation coefficient	Z value
Enterprise scale	0.604***	(26.54)
Asset-liability ratio	−1.711***	(−9.79)
Labor productivity	0.528***	(14.43)
Capital intensity	−0.063**	(−2.31)
Financing constraint	8.902***	(9.38)
Enterprise age	0.016***	(3.79)
Overseas business income	1.143***	(17.37)
Enterprise control attribute	−1.279***	(−17.69)
Industry effect	Yes	Yes
Time effect	Yes	Yes
N	24,946	
Pseudo-R	0.116	

***, **, * represent the significant level of 1, 5, 10%, respectively, and the numbers in parentheses are robust standard errors. Unless with specification, the following are the same.

According to the estimated results in Table 3, the coefficient of Size is significantly positive at the 1% level, which indicates that the larger the scale of enterprises, the greater the probability of overseas M&A. This is because these enterprises have more resources, have more strength to merge with other enterprises, can provide full play to the role of synergy and economies of scale, and can cope with all kinds of risks faced by overseas M&A. The coefficient of Lap is significantly positive at the 1% level, indicating that enterprises with higher labor productivity are more likely to produce overseas M&A. This is because these enterprises can overcome the investment barriers and information processing costs of the host country, which means that Chinese enterprises have a self-selective effect in the open innovation model based on overseas M&A (Mao et al., 2015), The coefficient of Age is significantly positive at the 1% level, indicating that the longer the establishment of enterprises, the higher the probability of overseas M&A. Simultaneously, the coefficient of Oversea is significantly positive at the 1% level, indicating that if enterprises have already carried out business overseas, they will be more likely to engage in overseas M&A. The fuller the understanding of the overseas market, the higher the business income and the more motivated the M&A of foreign companies, thus further improving the overseas market share. In addition, the uncertainty of information is also reduced, and the success rate of M&A is improved. The coefficient of Lev is significantly negative at the 1% level, which indicates that the higher the asset-liability ratio, the smaller the probability of overseas M&A. This is because enterprises with a high asset-liability ratio may face higher financial risks; thus, there are not enough self-owned funds to carry out overseas M&A. If overseas M&As are carried out, the financial risks they face expand further. It will even affect the normal business activities of the enterprise. The coefficient of Capital is significantly negative at the 5% level, which indicates that the higher the capital intensity, the smaller the probability of overseas M&A. The coefficient of Fc is significantly positive at the 1% level, which indicates that the greater the financing constraint, the easier it may be for enterprises with greater financing constraints to adopt a policy of radical expansion to carry out overseas M&A, so as to seek new technologies and resources to expand the market scale and their own business income. However, the State coefficient is significantly negative at the 1% level, which indicates that SOEs are not inclined to carry out overseas M&A. According to the data of the statistical bulletin of China’s foreign direct investment, the contribution of non-SOEs to cross-border M&A investment gradually exceeds that of SOEs, and occupies a major position in cross-border M&A.

Through the Logit model, it can be found that only those enterprises with productivity and technological advantages can carry out open innovation based on overseas M&A, which verifies that overseas M&A open innovation enterprises have a self-selection effect.

In order to solve this problem, according to the propensity score estimated by the Logit model, this study matches the control group enterprises closest to the experimental group in order to minimize the sample selection bias, and uses the k nearest domain matching method (*k* = 4, *r* = 0.001) to analyze the matching effect (In this study, different matching methods are used to obtain similar results); the premise of using the PSM model to match is to satisfy the parallel hypothesis and the common support hypothesis. Table 4 shows the test results of the control variables before and after matching. The results show that the *P*-value of all the matched (M) variables is greater than 0.1, and the overall LR (Likelihood Ratio. It is a kind of index reflecting authenticity, which is a composite index reflecting sensitivity and specificity at the same time) test shows that *P* = 1.000 after matching; thus, there is no significant difference between the experimental group and the control group, and the parallel trend hypothesis is satisfied.

**TABLE 4:** Results of equilibrium test using the PSM method.

Variable	Matching	Mean			% Reduced bias	T-test	
		Treated	Control	% Bias		*t*	*P* > *t*
Size	U	8.0156	7.3418	51.2		22.07	0.000
	M	8.0082	7.9905	1.3	97.4	0.40	0.687
Lev	U	0.4347	0.42002	7.2		3.04	0.002
	M	0.43417	0.42766	3.2	55.6	1..01	0.315
Lap	U	13.812	13.676	15.2		6.41	0.000
	M	13.808	13.814	–0.7	95.5	–0.21	0.836
Capital	U	12.321	12.301	1.6		0.66	0.507
	M	12.321	12.319	0.2	88.3	0.06	0.952
Fc	U	0.01646	0.01493	4.3		1.82	0.069
	M	0.01649	0.01577	2.0	52.3	0.65	0.514
Age	U	17.802	17.315	8.4		0.41	0.001
	M	17.794	17.797	–0.1	99.2	–0.02	0.984
Oversea	U	0.78136	0.5545	49.6		19.60	0.000
	M	0.7808	0.78337	–0.6	98.9	–0.19	0.846
State	U	0.20072	0.29	–20.9		–8.42	0.000
	M	0.20123	0.19995	–0.3	98.6	0.10	0.920

**FIGURE 2:**
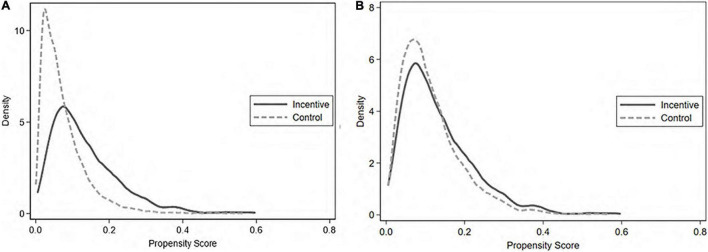
Kernel density maps **(A)** before and **(B)** after matching.

As shown in Figure 2, the kernel density distribution between the experimental and control groups is quite different before matching, but the kernel density distribution of the treatment and control groups tends to coincide after matching; thus, the common support hypothesis is verified. Therefore, this study has a good matching effect, and the choice of matching variables is more reasonable. The DID method can be further used to analyze the impact of open innovation based on overseas M&A on enterprise innovation.

### Results of DID

In this section, we conduct an empirical analysis to examine the impact of open innovation based on overseas M&A, including the full sample DID analysis and PSM-DID analysis.

#### The Results for the Full Sample

The regression results of the DID fixed effect are shown in Table 5. The four models are the influence of overseas M&A on enterprise innovation: (1) the total number of invention patents and utility model patents applied by enterprises in that year (Patent); (2) the number of invention patents applied by enterprises in that year (Invention); (3) the intensity of R&D investment (Rd); and (4) the proportion of technical personnel (Rdp). The results show that the regression coefficients of did are significantly positive at the 1% level, which indicates that overseas M&As can significantly improve the innovation performance and investment of enterprises. After overseas M&A, the quantity and quality of patent performance are significantly increased, and the intensity of R&D investment and the proportion of technical personnel are also significantly increased.

**TABLE 5:** DID regression results.

Variable	(1)	(2)	(3)	(4)
	Patent	Invention	Rd	Rdp
Did	0.513***	0.455***	0.006***	0.034***
	(9.84)	(9.30)	(5.67)	(6.93)
Size	0.582***	0.483***	–0.005***	–0.002***
	(61.69)	(55.81)	(–25.54)	(–2.71)
Lev	–1.283***	–1.029***	–0.019***	–0.123***
	(–22.25)	(–20.80)	(–13.14)	(–19.45)
Lap	0.268***	0.248***	–0.009***	0.014***
	(20.45)	(21.43)	(–23.80)	(9.64)
Capital	0.105***	0.070***	–0.001***	0.000
	(11.81)	(9.10)	(–5.07)	(0.22)
Fc	1.195***	1.074***	–0.079***	0.026
	(3.88)	(4.10)	(–7.86)	(0.73)
Age	0.021***	0.016***	–0.001***	0.001***
	(12.53)	(10.94)	(–17.12)	(7.95)
Oversea	0.421***	0.326***	0.008***	0.013***
	(20.53)	(18.67)	(14.42)	(5.71)
State	0.356***	0.308***	–0.001**	0.038***
	(14.30)	(13.86)	(–2.11)	(15.73)
Cons	–9.348***	–7.966***	0.198***	–0.106***
	(–41.81)	(–40.65)	(37.27)	(–4.80)
Industry effect	Yes	Yes	Yes	Yes
Time effect	Yes	Yes	Yes	Yes
Adj_R^2^	0.381	0.343	0.386	0.279
*N*	24,963	24,963	24,963	24,963
*F*	358.261	258.150	412.819	158.190

The t-value calculated based on the standard error of robustness is shown in brackets. ***, **, * represent the significant level of 1, 5, 10%, respectively.

#### The Results for PSM-DID

Table 6 shows the results of the PSM-DID regression. The four models are as follows: (1) the impact of overseas M&A on enterprise innovation, that is, the total number of invention patents and utility model patents; (2) the number of invention patents applied by enterprises in that year; (3) the R&D investment intensity (Rd); and (4) the proportion of technical personnel (Rdp). The results are shown in columns (1) to (4). The regression coefficients of Did are significantly positive at the 1% level, which indicates that after enterprises carry out open innovation based on overseas M&A, the quantity and quality of patent performance are significantly increased, and the intensity of R&D investment and the proportion of technical personnel are also significantly increased, indicating that open innovation based on overseas M&A can significantly improve the innovation performance and investment of enterprises. The high-quality development of the enterprise validates theoretical Hypothesis 1 of this study.

**TABLE 6:** Regression results of PSM-DID analysis.

Variable	(1)	(2)	(3)	(4)
	Patent	Invention	Rd	Rdp
Did	0.443***	0.365***	0.005***	0.037***
	(8.20)	(7.23)	(4.78)	(7.06)
Size	0.671***	0.584***	-0.005***	–0.010***
	(36.92)	(34.34)	(–15.56)	(–6.40)
Lev	–1.302***	–1.073***	–0.016***	–0.094***
	(–10.74)	(–9.99)	(–5.88)	(–7.71)
Lap	0.295***	0.273***	–0.010***	0.007***
	(11.27)	(11.45)	(–16.65)	(2.80)
Capital	0.116***	0.092***	–0.001**	–0.000
	(6.34)	(5.64)	(–2.56)	(–0.19)
Fc	2.030***	1.692***	–0.086***	–0.151**
	(3.22)	(3.10)	(–4.38)	(–2.27)
Age	0.017***	0.013***	–0.000***	0.002***
	(5.21)	(4.49)	(–6.76)	(6.04)
Overseas	0.407***	0.313***	0.009***	0.011**
	(9.02)	(7.97)	(9.63)	(2.18)
State	0.341***	0.344***	0.002**	0.035***
	(6.29)	(6.85)	(2.08)	(7.38)
Industry effect	Yes	Yes	Yes	Yes
Time effect	Yes	Yes	Yes	Yes
Adj_R^2^	0.415	0.378	0.375	0.310
*N*	7,691	7,691	7,691	7,691
*F*	140.149	104.820	134.711	54.432

Standard error is robust standard error. ***, **, * represent the significant level of 1, 5, 10%, respectively.

Since the influence of overseas M&A on enterprise innovation is not limited to that year, there may be a continuous impact in the following years. In order to better evaluate the impact of M&A, it is necessary to further investigate the dynamic effects, investigate the changes of innovation input and performance in the two years after M&A, and construct the following model:


(2) $$y_{i\,t}=\beta_{0}+\beta_{1}\,d\,i\,d_{0}+\beta_{2}\,d\,i\,d_{1}+\beta_{3}\,d\,i\,d_{2}+X_{i\,t}^{{}^{\prime }}\,\varphi +\eta_{j}+\gamma_{t}+\varepsilon_{i\,t}$$

Where *did*_0_,*did*_1_,*did*_2_ is a virtual variable, indicating the dynamic effects of the current year, the first year, and the second year after overseas M&A.

The empirical results, as shown in Table 7, show that the coefficient of *did*_0_*anddid*_1_ is significantly positive at the 1% level. The coefficient of *did*_2_ is significantly positive at the 5% level (at least), indicating that open innovation based on overseas M&A plays a significant role in promoting enterprise innovation performance and innovation investment compared with enterprises without overseas M&A. This promotion effect is sustainable, and in general, it shows a decreasing trend with the increase in years.

**TABLE 7:** Dynamic effect regression results.

Variable	(1)	(2)	(3)	(4)
	Patent	Invention	Rd	Rdp
did0	0.691***	0.589***	0.006***	0.057***
	(7.11)	(6.41)	(2.91)	(5.65)
did1	0.649***	0.517***	0.006***	0.034***
	(6.72)	(5.59)	(3.09)	(3.39)
did2	0.380***	0.330***	0.007***	0.025**
	(3.84)	(3.47)	(3.21)	(2.55)
Control variable	Control	Control	Control	Control
Industry effect	Yes	Yes	Yes	Yes
Time effect	Yes	Yes	Yes	Yes
Adj_R^2^	0.381	0.342	0.385	0.279
*N*	24,963	24,963	24,963	24,963

The control variables are the same as in Table 6 and are not fully listed for savings. The following is the same. ***, **, * represent the significant level of 1, 5, 10%, respectively.

The empirical results verify Hypothesis 1; that is, open innovation based on overseas M&A can significantly promote enterprise innovation. Enterprise innovation performance and innovation investment are significantly increased, and the role of promotion is sustainable. In the years of M&A, the promotion role reaches the maximum. Since then, it has shown a decreasing trend with the increase in years.

### Results of Heterogeneity Analysis

To verify Hypotheses 2 to 3 and explore the impact of enterprise ownership, and enterprise characteristics on the innovation effect of open innovation based on overseas M&A, a heterogeneity analysis is carried out by sample.

#### Heterogeneity Analysis of Enterprise Ownership

The empirical test results of Hypothesis 2 are presented in Table 8. It can be seen that when the patent quantity (Patent) and quality (Invention) are used as explained variables, the estimation coefficient of did is significantly positive at the 1% level, indicating that the ownership of ownership does not affect the promotion of open innovation based on overseas M&A on the innovation performance of enterprises; thus, there has been a significant improvement in both the quantity and quality of patent performance. Simultaneously, in terms of the number of patent performance (Patent), there is no significant difference in the promoting effect of open innovation based on overseas M&A on SOEs and non-SOEs. However, in terms of patent performance quality (Invention), the effect of open innovation based on overseas M&A on the improvement of patent quality of SOEs is obviously higher than that of non-SOEs. This means that SOEs absorb foreign advanced technology through overseas M&A, and promote their own R&D ability to obtain more obvious improvement, so that the number of invention patents is significantly increased.

**TABLE 8:** Heterogeneous regression results of enterprise ownership.

Variable	SOEs				Non-SOEs			
	(1) Patent	(2) Invention	(3) Rd	(4) Rdp	(5) Patent	(6) Invention	(7) Rd	(8) Rdp
Did	0.513***	0.575***	0.001	0.020**	0.520***	0.436***	0.008***	0.035***
	(4.05)	(4.59)	(0.91)	(2.02)	(4.07)	(8.26)	(6.69)	(6.14)
Control variable	Control	Control	Control	Control	Control	Control	Control	Control
Industry effect	Yes	Yes	Yes	Yes	Yes	Yes	Yes	Yes
Time effect	Yes	Yes	Yes	Yes	Yes	Yes	Yes	Yes
Adj_R^2^	0.478	0.435	0.345	0.258	0.331	0.290	0.359	0.304
*N*	7,065	7,065	7,065	7,065	17,898	17,898	17,898	17,898

The t-test showed that there was no significant difference in the coefficients of did between columns (1) and (5), while columns (2) and (6), columns (3) and (7), column (4), and column (8) had significant differences in the coefficients of did. ***, **, * represent the significant level of 1, 5, 10%, respectively.

When R&D investment (Rd) and human capital investment (Rdp) are used as explained variables, for SOEs, the estimation coefficient of did corresponding to R&D investment (Rd) was not significant, indicating that the overseas M&A of SOEs did not significantly affect the intensity of R&D investment, while the estimation coefficient of did corresponding to human capital investment (Rdp) was significantly positive at the 5% level. This shows that overseas M&As can significantly increase the proportion of technical personnel in SOEs. For non-SOEs, the estimation coefficients of did corresponding to R&D investment (Rd) and human capital investment were significantly positive at the 1% level, indicating that non-SOEs adopt the open innovation mode of overseas M&A to significantly increase the intensity of R&D investment and the proportion of scientific research personnel. Thus, the innovation investment of non-SOEs is significantly increased. It can be found from the coefficient that overseas M&A, an open innovation method, plays a much more important role in promoting the innovation investment of non-SOEs than those of SOEs. Through the open innovation model of overseas M&A, non-SOEs are more aware of the importance of technology and innovation, and are more willing to increase investment in capital and researchers. In summary, the empirical results support Hypothesis 2.

#### Heterogeneity Analysis of High-Tech Enterprises

In order to verify Hypothesis 4, the samples are divided into high-tech enterprises and non-high-tech enterprises, and the influence of open innovation based on overseas M&A on the innovation activities of M&A enterprises is studied. The empirical test results are shown in Table 9. It is found that when the patent quantity (Patent) and quality (Invention) are explained variables, the did coefficient is significantly positive at the 5% level (at least), and the corresponding coefficient of non-high-tech enterprises is much higher than that of high-tech enterprises. This means that overseas M&As play a significant role in promoting the innovation performance of high-tech enterprises and non-high-tech enterprises, the quantity and quality of patent applications are significantly increased, and the promotion of non-high-tech enterprises is stronger. This result supports Hypothesis 5.

**TABLE 9:** Heterogeneous regression results of high-tech enterprises.

Variable	High-tech enterprises				Non-high-tech enterprises			
	(1) Patent	(2) Invention	(3) Rd	(4) Rdp	(5) Patent	(6) Invention	(7) Rd	(8) Rdp
did	0.147**	0.159**	0.006***	0.025***	0.491***	0.447***	0.005***	0.032***
	(2.00)	(2.11)	(3.49)	(3.25)	(7.51)	(7.35)	(3.77)	(5.11)
Control variable	Control	Control	Control	Control	Control	Control	Control	Control
Industry effect	Yes	Yes	Yes	Yes	Yes	Yes	Yes	Yes
Time effect	Yes	Yes	Yes	Yes	Yes	Yes	Yes	Yes
Adj_R^2^	0.439	0.386	0.384	0.404	0.341	0.302	0.388	0.263
*N*	5912	5912	5912	5912	19051	19051	19051	19051

The t-test showed that there was no significant difference in the did coefficients between columns (3) and (7), columns (1) and (5), columns (2) and (6), and columns (4) and (8). There was a significant difference in the did coefficient between columns (1) and (5), columns (2) and (6), and columns (4) and (8). ***, **, * represent the significant level of 1, 5, 10%, respectively.

When R&D investment (Rd) and human capital investment (Rdp) are taken as explained variables, the coefficients of did corresponding to high-tech and non-high-tech enterprises are significantly positive at the 1% level, indicating that open innovation based on overseas M&A can promote the innovation investment of high-tech and non-high-tech enterprises. However, in terms of the regression coefficient, there is no significant difference in R&D investment. In the proportion of technical personnel, the open innovation mode based on overseas M&A plays a greater role in promoting non-high-tech enterprises than high-tech enterprises. This may be because the proportion of technical personnel in high-tech companies is inherently high; thus, the promotion effect brought about by M&A is not as obvious as that of non-high-tech companies.

In summary, for non-high-tech enterprises, open innovation based on overseas M&A plays a more obvious role in promoting the innovation activities of this type of enterprise. Specifically, the promoting effect on the quantity and quality of patent performance and the proportion of technical personnel is higher than that of high-tech enterprises, which means that for non-high-tech enterprises, the open innovation mode based on overseas M&A can obtain foreign technology. It is effective to improve the level of innovation, and such enterprises should be encouraged to go abroad.

## Discussion

Innovation is the first impetus that leads to development. The independent innovation behavior of enterprises is the first factor to achieve high-quality growth. More and more enterprises are using overseas M&A as the main way of open innovation to obtain external resources and promote innovation. However, the details of the impact of open innovation based on overseas M&A on enterprises’ independent innovation behavior are still in the black box. Therefore, the main objectives of this study were to empirically analyze the extent to which overseas M&A can enhance enterprises’ independent innovation behavior and examine the impact of enterprise ownership and enterprise characteristics on the innovation effect of overseas M&A.

In view of this, this paper focuses on the key variable of enterprise independent innovation behavior. Taking the overseas M&A of enterprises as a quasi-natural experiment, this paper uses the DID method to investigate the impact of open innovation based on overseas M&A on enterprises’ independent innovation behavior, and tests the robustness and heterogeneity. The empirical results answer the above questions well.

First, on the whole, on the basis of controlling other factors, open innovation based on overseas M&A can significantly promote enterprises’ innovation performance and innovation investment. This finding is consistent with previous studies’ conclusions that M&A enhances the innovation performance of enterprises (Yu et al., 2019; Cirjevskis, 2021), and provide empirical evidence that quantitatively answers the innovation effect of overseas M&A. In addition, through dynamic effect analysis, we found that this promotion effect of overseas M&A on enterprises’ independent innovation behavior is persistent. Specifically, this innovation effect reaches the maximum in the year of M&A, and then decreases in the next two years, but remains. This result is an important contribution to the academic literature because it not only provides empirical evidence for overseas M&A promote the independent innovation behavior of enterprises, but also shed light on the dynamics of this impact. This finding was lacking in previous studies. Based on this finding, when enterprises seek to enhance their innovation capabilities through external resources, overseas M&A is a recommended route. Enterprises should better put more attention on the first year after an overseas M&A, because the innovation effect is strongest in this year. The government should create a good M&A environment for enterprises, and encourage enterprises to conduct overseas M&A from the aspects of preferential tax policies, strengthening intellectual property protection, and broadening financing channels.

Second, the impact of open innovation based on overseas M&A on enterprise innovation is heterogeneous due to enterprise ownership, and technology intensity. In terms of enterprise ownership, open innovation based on overseas M&A has innovation effect for both SOEs and non-SOEs but different in the innovation performance and the innovation investment. To be specific, overseas M&A has a stronger promotion effect on the patent performance quality (Invention) among SOEs and the R&D investment (Rd) and human capital investment (Rdp) among non-SOEs. In terms of technology intensity, for non-high-tech enterprises, the promoting effect of open innovation based on overseas M&A on the quantity and quality of patent and the proportion of technical personnel is higher than that of high-tech enterprises. Previous studies provided a little discussion of the heterogeneity of the impact of M&A on innovative behavior of enterprises, which addressed that firm age have an important role in open innovation (Krishna and Jain, 2020). Our finding contributes to the academic literature since this result expands the understanding of the effects of open innovation based on M&A on innovative behavior of enterprises from the perspective of enterprise ownership and technology intensity, which are considered to be closely related to enterprise innovation (Yu et al., 2016; Liu et al., 2017; Shen et al., 2019). Based on this finding, overseas M&A enterprises should also increase R&D intensity and efficiency, cultivate innovative talents by various ways, and build a competitive innovation system, which can not only improve the success rate of overseas M&A transactions, but also promote the technology complementarity and integration among enterprises (Wang and Liu, 2018), and benefit from the open innovation mode based on overseas M&A to a greater extent. The government should formulate more detailed and targeted support policies for overseas M&A, create favorable conditions for open innovation and cooperation among enterprises, universities, colleges and other institutions, and guide various types of enterprises’ open innovation behavior based on overseas M&A.

## Conclusion

Taking the results of the study into account, this research make several contributions to the existing literature. First, this study uses the relevant data of Chinese listed companies from 2011 to 2018 to empirically study the causal relationship between the open innovation of listed companies based on overseas M&A and enterprise independent innovation behavior. Second, in the research method, the overseas M&A of listed companies is regarded as a quasi-natural experiment, and the DID and PSM method are used to solve the self-selection bias of samples and reduce the endogenous problem, which makes the causal identification of this paper clearer in a certain process. Third, this study contributes to a comprehensive understanding of the innovation effects of open innovation based on overseas M&A, as this study considers both the innovation performance and investment of enterprises and further analyzes the heterogeneous innovation effect of open innovation based on overseas M&A among different enterprise ownership and technology intensity.

This study is not without limitations and future work may explore the following issues. First, this study uses the data of listed companies and lacks an examination of the relationship between overseas M&A and innovation in small and medium-sized enterprises. Second, only Chinese list companies are considered in this study; due to differences in national policies and stages of development, using data of companies from other countries to answer this question would make this study more robust. Third, in the process of M&A, through the outflow, inflow and integration of knowledge, the acquired company also engages in open innovation. It would be interesting to compare whether the result would be the same for acquired company in future research.

## Data Availability Statement

Publicly available datasets were analyzed in this study. This data can be found here: State Intellectual Property Office, www.cnipa.gov.cn; Wind database, www.wind.com.cn; China Stock Market & Accounting Research Database (CSMAR), www.gtarsc.com.

## Author Contributions

MW led and designed the study, led the data collection, analysis, and interpretation. TL contributed to the study design, provided input into the data analysis, and wrote the first draft of the manuscript. YT contributed to the study design, reviewed the manuscript and helped the writing of the final draft manuscript. All authors read and approved the final manuscript.

## Conflict of Interest

The authors declare that the research was conducted in the absence of any commercial or financial relationships that could be construed as a potential conflict of interest.

## Publisher’s Note

All claims expressed in this article are solely those of the authors and do not necessarily represent those of their affiliated organizations, or those of the publisher, the editors and the reviewers. Any product that may be evaluated in this article, or claim that may be made by its manufacturer, is not guaranteed or endorsed by the publisher.

## Abbreviations

M&A: mergers and acquisitions
PSM: propensity score matching
DID: difference-in-difference
SOEs: state-owned enterprises.
